# Effects of spironolactone in heart failure with preserved ejection fraction: A meta-analysis of randomized controlled trials

**DOI:** 10.1097/MD.0000000000011942

**Published:** 2018-08-21

**Authors:** Shuai Li, Xinling Zhang, Mei Dong, Shu Gong, Zhi Shang, Xu Jia, Wenqiang Chen, Jianmin Yang, Jifu Li

**Affiliations:** a Key Laboratory of Cardiovascular Remodeling and Function Research, Chinese Ministry of Education and Chinese Ministry of Health, Department of Cardiology, Qilu Hospital of Shandong University; b The Heart Center, Jining First People's Hospital, Jining, Shandong, China.

**Keywords:** heart failure with preserved ejection fraction, meta-analysis, spironolactone

## Abstract

**Background:**

Heart failure with preserved ejection fraction (HFpEF) is a common syndrome, accounting for more than one half of all heart failure patients, which is associated with high morbidity and mortality. But there is little evidence-based therapeutic strategies for the management of HFpEF. Previous studies reported the effects of spironolactone on HFpEF; however, the results were inconsistent. In this meta-analysis, we evaluated the effects of spironolactone on HFpEF.

**Methods:**

Articles were searched on PubMed, EMBASE, and COCHRANE databases before May, 2017, and were supplemented by hand searches of reference lists of included studies and review articles. Eligible articles were restricted to randomized controlled trials (RCTs). The odds ratios (ORs) of the dichotomous data, mean difference (MD) of continuous data, and 95% confidence intervals (CIs) were calculated to assess the effects of spironolactone in patients with HFpEF.

**Results:**

A total of 7 studies including 4147 participants were analyzed. There were significant improvements on the E/e′ index (MD −1.38; 95% CI, −2.03 to −0.73; *P* < .0001) and E/A velocity ratio (MD −0.05; 95% CI, −0.10 to −0.00; *P* = .03) under spironolactone treatment compared with placebo, while there was no effect on the deceleration time (MD 1.04; 95% CI, −8.27 to 10.35; *P* = .83). Subgroup analyses on the E/A velocity ratio showed that there was obvious benefit from spironolactone therapy in patients with follow-up periods >6 months but not in those with follow-up periods ≤6 months. There was no reduction in all-cause mortality and hospitalization compared with placebo. And no improvement in 6-minute walk distance was seen compared with placebo.

**Conclusion:**

This meta-analysis demonstrates that the use of spironolactone improves left ventricular diastolic function in patients with HFpEF, whereas it has no effect on all-cause mortality and hospitalization, and the 6-minute walk distance. Further larger size, multicenter, RCTs are required to confirm the effects of spironolactone on patients with HFpEF.

## 1 Introduction

Heart failure with preserved ejection fraction (HFpEF) accounts for nearly half of all heart failure patients, and the proportion of patients with HFpEF increases with age.^[1–3]^ Patients with HFpEF are more likely to be female, older, obese, and have hypertension, atrial fibrillation, or diabetes mellitus.^[4–6]^ The survival rate of patients with HFpEF is not higher than that of patients with heart failure with reduced ejection fraction (HFrEF).^[7]^ In the past decades, the prognosis of HFpEF patients has not been improved, whereas that of patients with HFrEF has been achieved. What is more, no affirmative treatment has been proven effective at present in improving the survival rate in HFpEF patients. Now the treatment for HFpEF remains empirical and still lacks evidence-based therapeutic strategies. However, the role of spironolactone in HFpEF treatment remains unclear, which has been proved beneficial for HFrEF patients. We therefore conducted this meta-analysis by summarizing available randomized controlled trials (RCTs) related to spironolactone and HFpEF in order to assess the efficacy of spironolactone in HFpEF patients.

## 2 Materials and methods

### 2.1 Search strategy

We searched the electronic databases, PubMed, EMBASE, and the Cochrane Library for Central Register of Clinical Trials before May 2017, using the following search terms: “heart failure with preserved ejection fraction” or “heart failure with normal ejection fraction” or “diastolic heart failure” or “diastolic dysfunction” or “preserved cardiac function heart failure” or “HFpEF,” and “spironolactone.” We limited our search to studies in human subjects and English language. The articles were supplemented by hand searches of reference lists of included studies and review articles. We also searched one meta-analysis published previously on mineralocorticoid receptor antagonists and their relevant references.^[8]^

### 2.2 Inclusion and exclusion criteria

Eligible studies had to meet the following inclusion criteria: RCT; HFpEF was defined as signs or symptoms of heart failure with an left ventricle ejection fraction (LVEF) ≥45%; assessment of the effects of spironolactone on HFpEF; studies reporting end points for mortality, hospitalization, diastolic function (such as early to late diastolic transmitral flow velocity [E/A velocity ratio], ratio of mitral inflow early diastolic velocity to peak early diastolic mitral annular velocity [E/e′ velocity ratio], E wave deceleration time [DT]), or 6-minute walk distance (6MWD); and clearly defined intervention and control groups. The exclusion criteria were: healthy persons enrolled in the control group; deficiency of quantitative description of endpoints; patients with heart transplantations; and trials in the abstract form without a published manuscript. In addition, review, case report, and studies that had duplicated data were excluded.

### 2.3 Data extraction and study quality assessment

Data extraction was performed by 2 independent authors (SL and SG) using a standardized data collection form. All discrepancies were resolved through discussion between the 2 authors or by the 3rd reviewer (ZS). For every study, the first author, year of publication, sampling size, dose and treatment duration of spironolactone, cut off of LVEF for HFpEF, characteristics of study patients (such as age, sex, percentages of hypertension and diabetes mellitus, proportion of coronary artery disease, and atrial fibrillation), New York Heart Association class, and outcome data (including all-cause mortality, hospitalization, diastolic function and 6MWD) were extracted. The quality of each included randomized controlled study was evaluated by the modified Jadad quality scale,^[9]^ which evaluated studies based on adequate randomization, concealment of allocation, double blinding, and differential losses to follow-up or dropouts per treatment group. Studies with a score ≥4 were defined as high quality, and which with a score ≤3 were defined as low quality.

### 2.4 Outcomes assessed

We compared the outcomes between the group of patients who received spironolactone treatment and those who did not (control group). The primary outcome was the diastolic function, such as the E/A velocity ratio, E/e′ index, and E wave DT. The secondary outcome was the clinical events, such as all-cause mortality and hospitalization. The third outcome was the 6MWD.

### 2.5 Statistical analysis

All analyses were performed by the Cochrane Collaboration Review Manager 5.3. When the *P* value was less than .05, the difference between experimental groups and control groups was considered statistically significant. The odds ratio (OR) of dichotomous data (the secondary outcome), mean difference (MD) of continuous data (the primary outcome and the third outcome), and 95% confidence interval (95% CI) were separately calculated for each study. We used a random effect model (Der Simonian Laird) rather than a fixed-effect model, because it takes heterogeneity between multistudies into account. Statistical heterogeneity of studies in the meta-analysis was performed by the value of *I*^2^, which is defined as the percentage of overall differences among studies that is due to heterogeneity rather than chance.

When heterogeneity existed, which was considered as the *I*^2^ statistic more than 50%,^[10]^ subgroup analysis and sensitivity analysis were conducted to explore the causes of heterogeneity. In addition, subgroup analysis, including dosage of spironolactone, age, follow-up of spironolactone treatment, and cut-off of LVEF, were conducted on the E/A velocity ratio in order to investigate the effects of different subgroups on the outcome. We did not perform subgroup analysis on other outcomes because of the lack of sufficient data.

### 2.6 Ethical review

This study was a meta-analysis, which was based on the published data, thus no ethical approval was required.

## 3 Results

This meta-analysis was performed according to the Preferred Reporting Items for Systematic Reviews and Meta-analyses (PRISMA) statement for RCTs.^[11]^

### 3.1 Study selection

**Figure 1:**
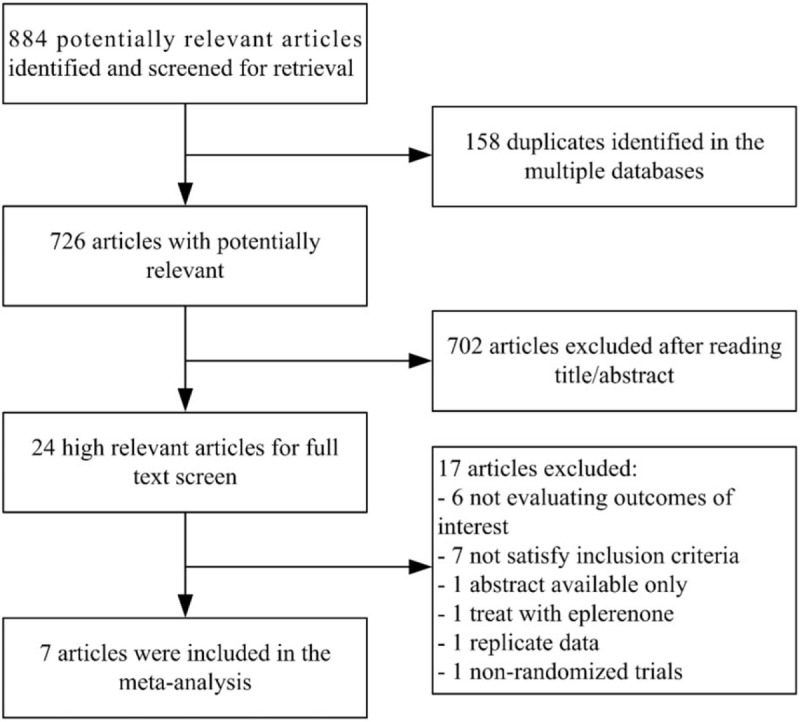
Preferred Reporting Items for Systematic Reviews and Meta-Analyses (PRISMA) flow diagram of study selection.

We screened 884 potentially relevant articles through 3 databases, in which there were 158 duplicates. After reading the titles and abstracts, 702 articles were excluded. Twenty-four articles were considered to be highly relevant and were screened for full text. By reading the full text, 17 articles were excluded for various reasons: 3 articles described other aspects of the TOPCAT trial, 3 articles did not contain our outcomes of interest, 2 articles included patients with LVEF <45%, 4 articles included patients who had only diastolic dysfunction without heart failure, 1 article described chronic heart failure without mentioning LVEF of included patients, 1 article had only available abstract, 1 article used eplerenone treatment as experimental group, 1 article had replicate data, and 1 article was a retrospective study. Ultimately, 7 studies^[12–18]^ satisfied the inclusion criteria. It is important to point out that the study carried by Shah AM evaluated the diastolic function of some people in the study carried by Pitt B. A flow diagram of study selection is presented (Fig. 1).

### 3.2 Baseline characteristics

The overall characteristics of the included 7 randomized clinical trials with a total of 4147 patients in this meta-analysis are listed in Table 1. The follow-up of treatment varied from 6 to 39.6 months. The mean age of study population ranged from 62 to 71 years. The patients were female predominately, and the dosage of spironolactone was 25 mg/day dominantly. Study population ranged from 30 to 3445 patients. Five studies defined HFpEF as LVEF ≥50%, another 2 defined HFpEF as LVEF ≥45%. Among them, diastolic function as the outcome was observed in 6 studies, clinical events as the outcome were reported in 2 studies, 6MWD was measured in 3 studies.

**Table 1:**
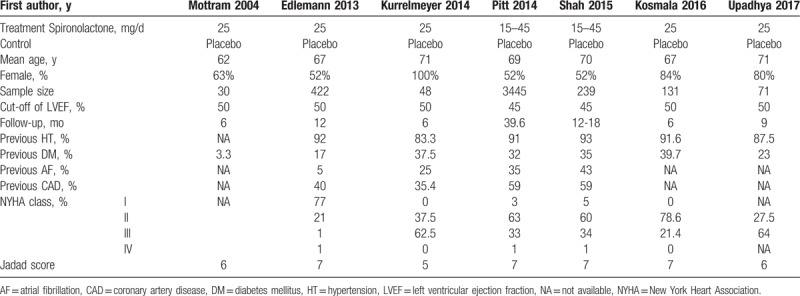
Characteristics of included studies.

### 3.3 Quality assessment

Various tools are designed for performing study quality assessment, the Jadad score is frequently used to assess the quality of RCTs. In this meta-analysis, a modified Jadad score was used to assess the quality of the 7 articles included. The scores of all included studies were ≥5, so all of them were deemed to have high quality (Jadad score ≥4). The Jadad score of each study was summarized in Table 1. We use the value of *I*^2^ to evaluate the heterogeneity of the studies. The *I*^2^ statistic was ≤50% in every outcome; therefore, we can consider that there was no significant heterogeneity in our meta-analysis.

### 3.4 Diastolic function

**Figure 2:**
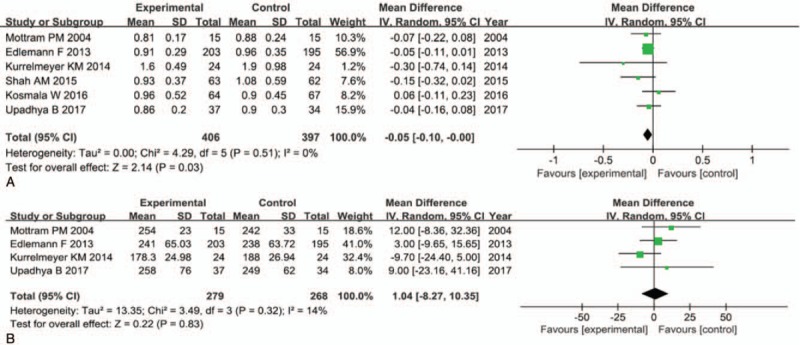
Forest plots of the E/A velocity ratio and DT between spironolactone group (experimental group) and control group. DT = deceleration time, E/A velocity ratio = ratio of early to late mitral flow.

**Figure 3:**
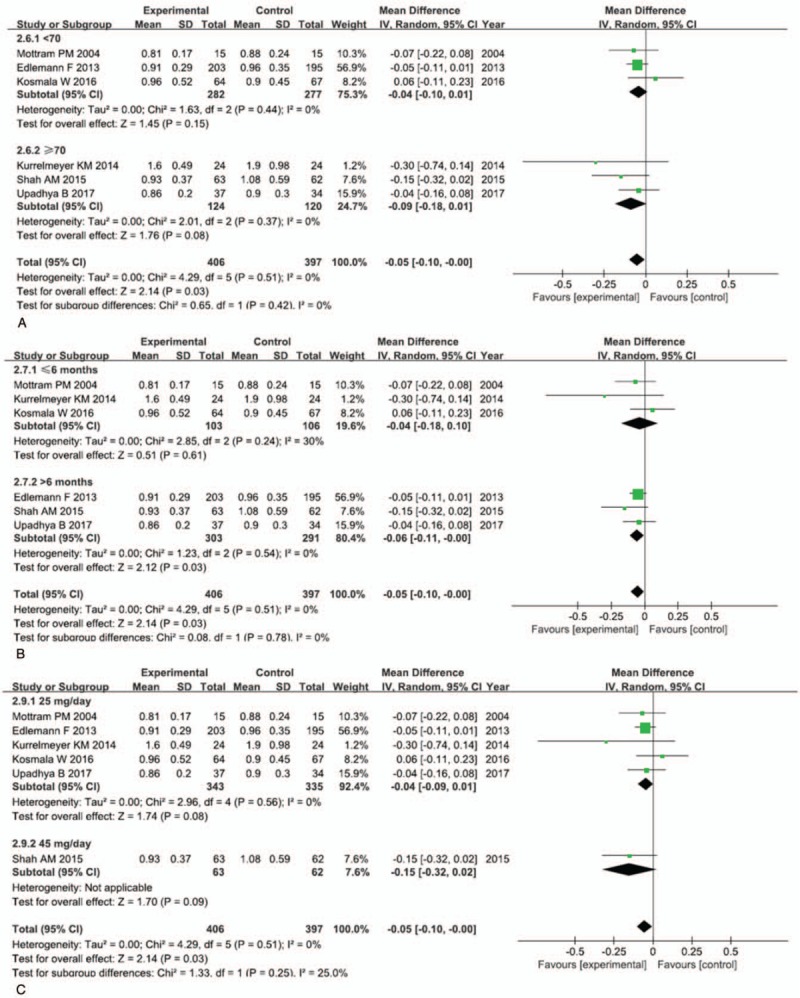
Forest plots of subgroup analyses on the ratio of early to late mitral flow (E/A velocity ratio). (A) The effect of age on the E/A velocity ratio, (B) the effect of follow-up period on the E/A velocity ratio, and (C) the effect of dosage of spironolactone on the E/A velocity ratio.

Data on the E/A velocity ratio were available from 6 trials, with 732 patients (406 in the experimental group and 397 in the control group) included in the analysis. We pooled the whole data to process, and found that there was an improvement on the E/A velocity ratio (MD −0.05; 95% CI, −0.10 to −0.00; *P* = .03) in the spironolactone group (Fig. 2A). Although the upper limit of the CI was 0, we still deemed that there were statistically significant differences between the 2 groups, because of the *P* = .03. In the future, we need to include more studies for this outcome. Subgroup analysis (Fig. 3) for the E/A velocity ratio showed that patients in the spironolactone group with follow-up periods more than 6 months (MD −0.06; 95% CI, −0.11 to −0.00, *P* = .03) had significant benefits, compared with patients whose follow-up periods was less than 6 months (MD −0.04; 95% CI, −0.18 to 0.10; *P* = .61). But subgroup analysis evaluating on mean age of included patients and dose of spironolactone did not have significant differences between the 2 groups. Only 2 studies described the E/e′ index, and pooling the results of them found a significant reduction in the E/e′ (MD −1.38; 95% CI, −2.03 to −0.73; *P* < .0001) after spironolactone treatment (Table 2). There was no significant change on DT with the use of spironolactone (MD 1.04; 95% CI, −8.27 to 10.35; *P* = .83) (Fig. 2B).

**Table 2:**
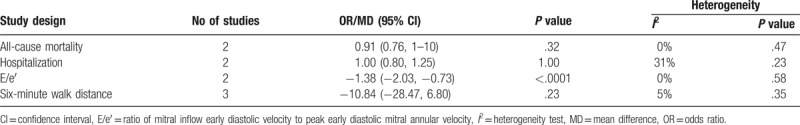
Analyses of other outcomes.

### 3.5 Clinical outcomes

There were 2 studies relating to all-cause mortality and hospitalization. Pooling results of the studies did not show any significant reduction in all-cause mortality rates (OR 0.91; 95% CI, 0.76–1.10; *P* = .32) and hospitalization rates (OR 1.00; 95% CI 0.80–1.25; *P* = 1.00) without obvious heterogeneity (Table 2). There was only 1 study known as TOPCAT trial^[15]^ referring to hospitalization of heart failure, which resulted in no sufficient data to analyze.

### 3.6 6MWD

Three studies with 519 patients related to 6MWD. There was no significant differences (MD −10.84; 95% CI, −28.47 to 6.80; *P* = .23) between spironolactone group and control group without obvious heterogeneity (Table 2).

## 4 Discussion

To our knowledge, our meta-analysis is the first study to evaluate the effects of spironolactone in patients with HFpEF by pooling data from related RCTs. This meta-analysis indicates that spironolactone therapy can improve diastolic function in HFpEF. Moreover, this improvement is significant in patients with longer follow-up periods. However, improvements in clinical outcomes and 6MWD are not significantly demonstrated according to the pooled results.

In our meta-analysis, pooled analysis of 2 trials mentioning clinical end points showed no reduction by spironolactone treatment. Notably, our study contained TOPCAT,^[15]^ which was a multicenter, international, RCT of spironolactone in HFpEF recruited from 6 countries. This large RCT showed neutral results. However, in TOPCAT subgroup analysis,^[19]^ the rates of the primary outcome (cardiovascular death, and hospitalization for heart failure) were significantly reduced by spironolactone in the Americas with no significant effects in Russia/Georgia. Therefore, regional and racial heterogeneity should be considered. None of other trials reported specific region and race of enrolled patients, consequently, we are unable to carry out subgroup analysis on geographical areas. Repeating the meta-analysis with regional and racial data of individual patient will provide greater insights in different regional and racial population.

The definition and diagnosis of HFpEF are still not universally accepted. According to 2016 ESC Guidelines,^[20]^ heart failure is divided into 3 broad categories based on measurement of the LVEF: patients with an LVEF ≥50% are defined as HFpEF, patients with an LVEF <40% are defined as HFrEF, and patients with an LVEF in the range of 40% to 49% are defined as heart failure with mid-range ejection fraction (HFmrEF). The diagnosis of HFpEF requires meeting the following conditions^[20]^: symptoms and/or signs of HF; LVEF ≥50%; elevated BNP or NT-proBNP; and relevant structural heart disease (left ventricular hypertrophy and/or left atrial enlargement) or diastolic dysfunction. But following 2017 ACC Guidelines,^[21]^ heart failure is divided into 2 categories based on measurement of the LVEF: patients with an LVEF >40% are defined as HFpEF, and those with an LVEF ≤40% are defined as HFrEF. In the HFpEF population, existing RCTs have used various LVEF cut-offs, ranging from 40% to 50%. In our meta-analysis, we define HFpEF as LVEF ≥45%. Therefore, data summarized in this study include patients with HFmrEF.

The pathophysiology of HFpEF is complicated, which is characterized by increased myocardial fibrosis and stiffness and impaired LV relaxation.^[22,23]^ Most patients with HFpEF have a high LV mass ratio and concentric remodeling,^[24]^ accompanied by cardiomyocyte hypertrophy. And HFpEF patients have interstitial fibrosis and extracellular matrix changes, which result in myocardial stiffness and reduced left ventricular filling,^[25–27]^ subsequently diastolic dysfunction occurs. Aldosterone is a complex steroid hormone having widespread physiologic effects that can act on multiple organs. Within the cardiac muscle, aldosterone binding to mineralocorticoid receptors promotes extracellular matrix and collagen deposition that stimulates myocardial fibrosis.^[28–31]^ Spironolactone, a nonselective mineralocorticoid receptor antagonists, can prevent myocardial fibrosis.^[28,32,33]^ Since spironolactone prevents cardiac fibrosis, it may be beneficial in HFpEF. Kosmala et al^[34,35]^ noticed that spironolactone significantly improved E/e′ velocity ratio, but had no effect on E/A velocity ratio and DT. Nevertheless, in our meta-analysis, except for E/e′ velocity ratio and E/A velocity ratio, the rest of outcomes show no benefit from the treatment of spironolactone in HFpEF. On the one hand, it may be because numbers of trials enrolled is low, which has consequences for the interpretation of the data and may narrow the impact of spironolactone on patients suffering from HFpEF. On the other hand, the treatment time is too short to benefit from the treatment of spironolactone. In the subgroup analysis stratified by follow-up periods, we found that trials with longer follow-up demonstrated greater reductions in E/A velocity ratio compared with shorter trials. As we all know that HFpEF is a chronic disease, a long-term treatment is needed. In addition, subgroup analysis stratified by mean age showed that there was no significant difference between older patients (70 years of age or older) and younger patients (less than 70 years old). Of note, the *P* value of older patients was smaller than that of younger patients. It was contrary to the study conducted by Zhang et al^[36]^ which mentioned that younger patients with HFpEF might obtain benefits from renin angiotensin aldosterone system inhibitors. Although subgroup analysis on dose of spironolactone revealed that there was no significant difference between lower dose (25 mg/day) and higher dose (45 mg/day) of spironolactone, further study is needed to explore the appropriate dose of spironolactone for HFpEF.

Notably, there are many indicators for evaluating diastolic function, in which most studies often adopt E/A, E/e′ velocity ratio, and DT as the main index. According to 2016 recommendations for the Evaluation of Left Ventricular Diastolic Function by Echocardiography,^[37]^ 4 variables are recommended: annular e’ velocity, average E/e’ ratio, LA maximum volume index, and peak tricuspid regurgitation velocity. Average E/e′ ratio is recommended for simplification and accuracy while E/A ratio is susceptible to age and the combined subclinical disorders. When it comes to our meta-analysis, spironolactone can significantly reduce E/e′ ratio and E/A ratio, so there are reasons to believe that spironolactone can improve diastolic function in HFpEF patients.

6MWD was put to indicate exercise capacity in our meta-analysis. There was no significant effect of spironolactone for HFpEF patients compared with the placebo group. Besides 6MWD, peak oxygen uptake and exertional E/e′ ratio were also taken as indicators describing exercise capacity, which we did not analysis because of insufficient data of enrolled studies. Trial conducted by Kosmala et al^[17]^ is contrary to that conducted by Upadhya^[18]^ about exercise capacity in HFpEF patients. It is possible that the selection of individual suffering from HFpEF is discrepant, because HFpEF is a very heterogeneous disease. The mechanisms of exercise intolerance in patients with HFpEF are not well understood. It may be because that increased diastolic stiffness prevents the increase in stroke volume during exercise, therefore, patients with HFpEF experience dyspnea and fatigue.^[38]^ In our meta-analysis, spironolactone can improve diastolic function in HFpEF patients while it has no impact on exercise capacity. Maybe it is limited to short-term follow-up periods and small numbers of trials evaluating exercise capacity, which lead to an insufficient power to detect the true effect of spironolactone.

## 5 Limitations

Our meta-analysis has several limitations. First of all, as mentioned above, HFpEF is defined as LVEF ≥50% according to 2016 ESC Guidelines. RCTs have used various LVEF cut-offs before 2016, ranging from 40% to 50%. In this meta-analysis, we used LVEF ≥45% as inclusion criteria. As a result, pooled analysis inevitably included patients with HFmrEF. Second, we assess the effects of spironolactone in HFpEF population from three aspects: diastolic function, clinical outcomes, and 6MWD. But those outcomes are not simultaneously included in each study. Therefore, there may be a mix of studies including different endpoints. Third, numbers of studies included are low, which may affect the consequences of the pooling data. In the future, more researches included may lead to more convincing results. Finally, half of studies that treatment duration are ≤6 months, but HFpEF is a chronic disease, it is important to know whether spironolactone actions persist for a much longer period. In our subgroup analysis, there is improvement in a longer spironolactone treatment duration in diastolic function. We call for further studies with a longer follow-up duration to discuss the effects of spironolactone on HFpEF patients.

## 6 Conclusion

In conclusion, our meta-analysis suggests that the use of spironolactone can improve diastolic function in patients with HFpEF and further study is needed to explore the appropriate dose of spironolactone. Further adequately powered studies are needed to confirm the effects of spironolactone on patients with HFpEF.

## Author contributions

**Data curation:** Zhi Shang, Shu Gong.

**Methodology:** Xu Jia.

**Resources:** Jianmin Yang.

**Software:** Shuai Li.

**Supervision:** Jifu Li.

**Visualization:** Xinling Zhang, Wenqiang Chen.

**Writing – original draft:** Shuai Li, Mei Dong.

**Writing – review & editing:** Xinling Zhang.
